# Supplementary material for: Proviral NUP153 binding to viral proteins and RNA regulates structural–nonstructural protein ratios in orthoflavivirus infection
Source: Nat Commun. 2026 Apr 8;17:3402. doi: 10.1038/s41467-026-71449-1 (PMC13068965; doi:10.1038/s41467-026-71449-1)
Supplement: Supplementary file 1 — Supplementary Information [file 41467_2026_71449_MOESM1_ESM.pdf]

Supplementary information

Proviral NUP153 binding to viral proteins and RNA regulates structural–nonstructural protein ratios in orthoflavivirus infection

Marie B. A. Peters[1,2], Richard Lindqvist[1,2], Eszter Kassa[3], Wai-Lok Yau[2,4], Pallabi Sengupta[5], Isabell Niedermoser[6], Gisa Gerold[6], Nasim Sabouri[5,7], Ylva Ivarsson[3], Richard Lundmark[2,4] & Anna K. Överby[1,2*]

[1]Department of Clinical Microbiology, Umeå University, Umeå, Sweden

[2]Laboratory for Molecular Infection Medicine Sweden (MIMS), Umeå University, Umeå, Sweden

[3]Department of Chemistry for Life Sciences, Uppsala University, Uppsala, Sweden

[4]Department of Medical and Translational Biology, SciLifeLab, Umeå University, Umeå, Sweden

[5]Department of Medical Biochemistry and Biophysics, Umeå University, Umeå, Sweden

[6] Institute of Virology, Medical University of Innsbruck, Innsbruck, Austria

[7] Science for Life Laboratory, Umeå University, Umeå, Sweden

*Corresponding author: Anna K Överby

Email: anna.overby@umu.se

Supplementary Figure and figure legends 1-8

Supplementary Tables 1-2

Supplementary Figures

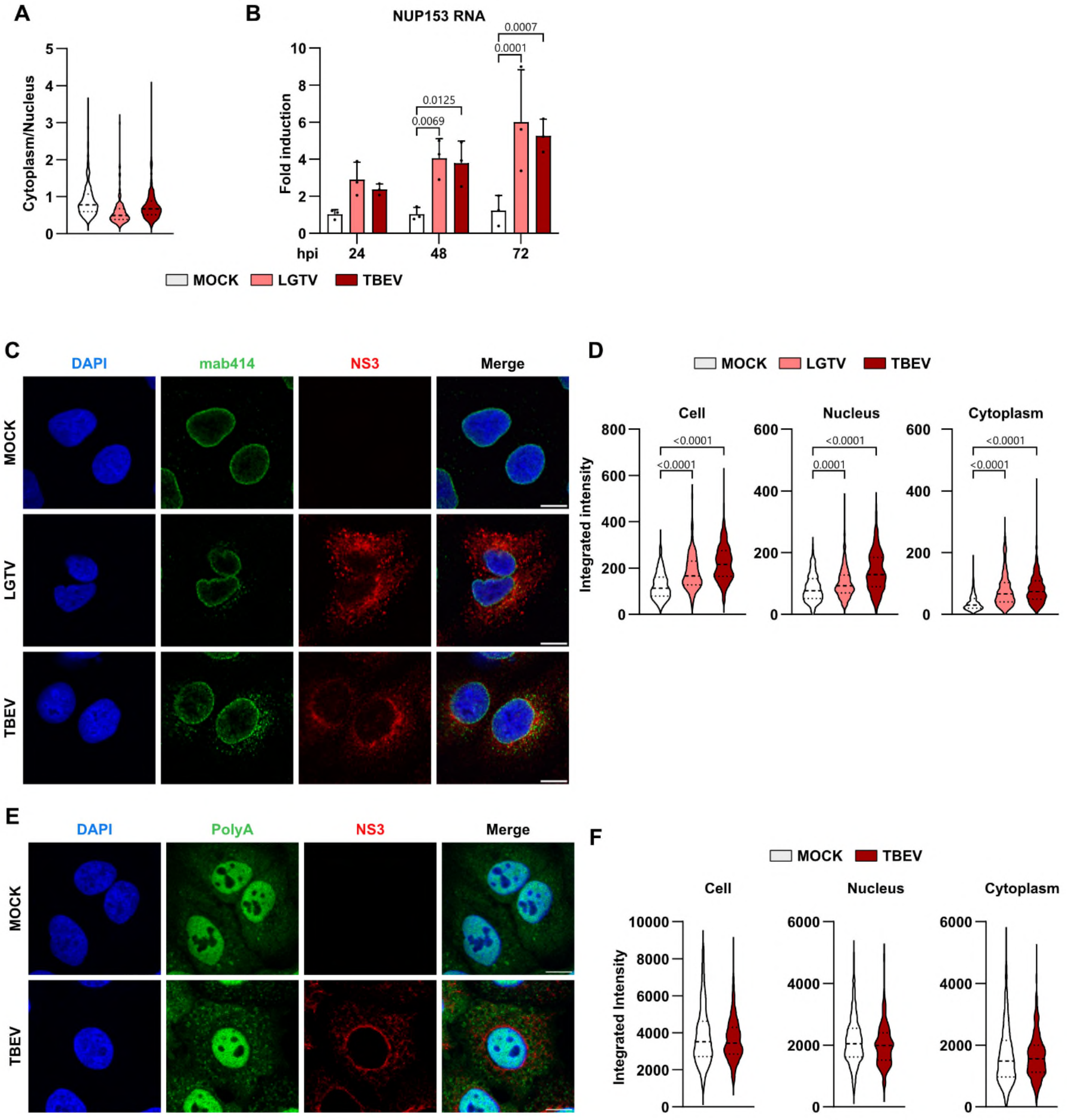

Supplementary Figure 1: NUP153 is upregulated during TBEV and LGTV infection without affecting NPC function. (A) Quantification of NUP153 ratio between cytoplasm and nucleus of A549 cells infected with LGTV and TBEV for 24 h. **(B)** Fold induction in NUP153 RNA levels in HEK293T cells infected with LGTV or TBEV at 24, 48 and 72 hpi using RT-qPCR. Samples were normalized to actin RNA and mock treated samples of each timepoint. Data presented as mean+SD (n=3 biological replicates). P values calculated two-way ANOVA

using Dunnett's multiple comparison test. **(C-D)** Representative confocal image of A549 cells infected 24 h with LGTV and TBEV. Cells were stained with DAPI (blue), anti-NS3 (red) and anti-mab414 (green). Scale bars, 10 µm. **(D)** Quantification of Mab414 antibody integrated intensity in different cell segments. Data are presented as violin plot (n=335 [Mock], n=272 [LGTV], and n=315 [TBEV]). P values calculated by one-way ANOVA using Kruskal–Wallis test. **(E)** FISH analysis of A549 cells infected 24 h with TBEV. Cells were stained with DAPI (blue), poly-T probes (green) and anti-NS3 (red); and imaged by confocal microscopy. Scale bars, 10 µm. **(F)** Quantification of PolyA integrated intensity in different cell segments. Data are presented as violin plot (n=677 [Mock], and n=367 [TBEV]).

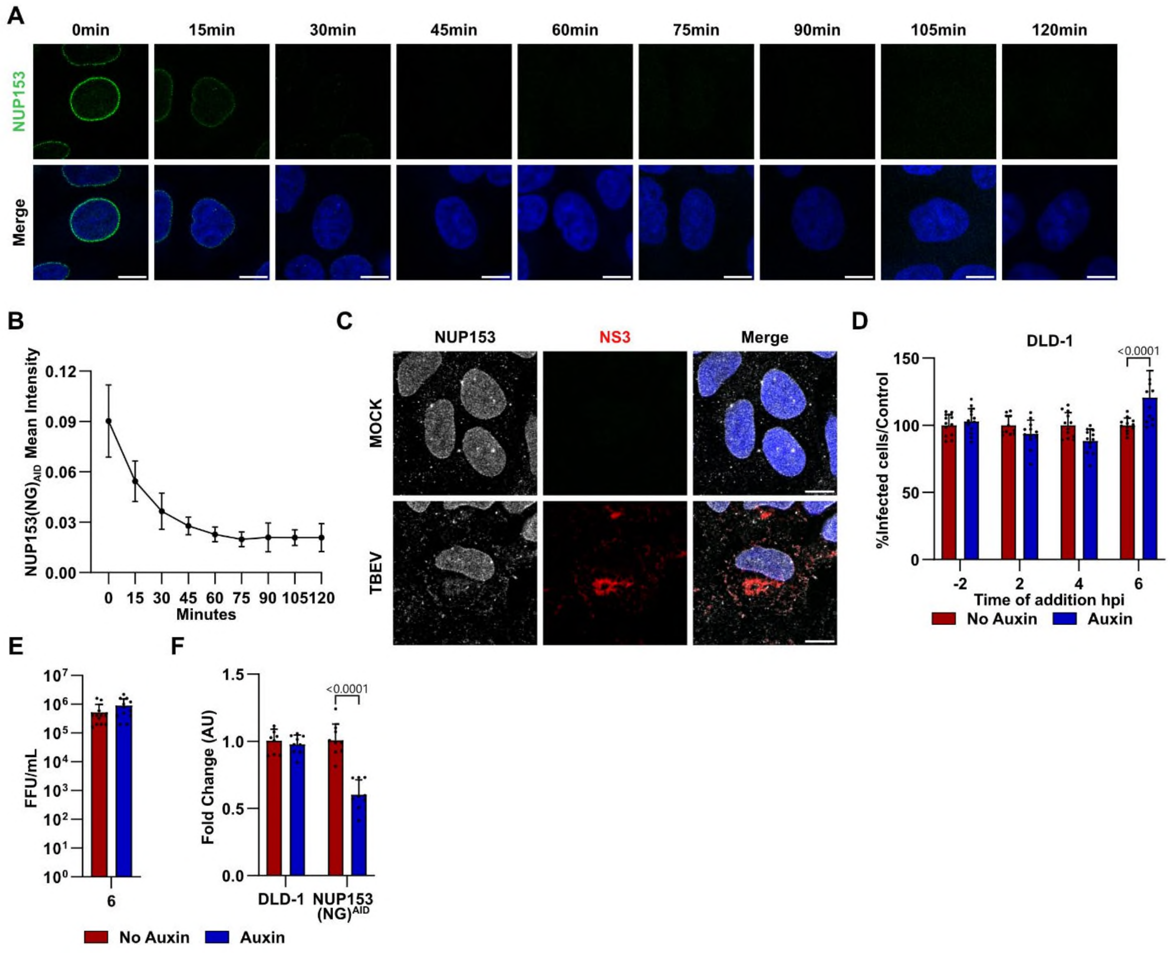

Supplementary Figure 2: NUP153 is a proviral factor during TBEV infection. (A-B) Immunofluorescence analysis using confocal microscopy of NUP153(NG)^{AID} (green) degradation in NUP153(NG)^{AID} cells over time following auxin induction. NUP153(NG)^{AID} (green) and DAPI (blue). Scale bars, 10 µm. **(B)** Quantification of NUP153(NG)^{AID} mean intensity over time following auxin induction. Data plotted as mean±SD (n=272 [0 min], n=237 [15 min], n=245 [30 min], n=272 [45 min], n=145 [60 min], n=125 [75 min], n=204 [90 min), n=53 [105 min], and n=180 [120 min]). **(C)** Immunofluorescence assay of DLD-1 cells infected 24 h with TBEV. Cells were stained with DAPI (blue), anti-NUP153 (greys) and anti-NS3 (red). Scale bars, 10 µm. **(D)** Auxin time of addition experiment on DLD-1 cells infected with TBEV 16 h. Quantification of E-positive cells normalized to uninduced cells analyzed by plate reader. n=12 from 3 biological replicates and error bars represent means+SD. P value calculated with two-way ANOVA using Šídák's multiple comparisons test. **(E)** Quantification of viral titers

NUP153(NG)^{AID} cells infected with TBEV 16 hours, following auxin induction (1 mM) at 6 hpi. Data presented as mean+SD (n=12 from 3 biological replicates). (**F)** Quantification of TBEV RNA in DLD-1 and NUP153(NG)^{AID} cells infected with TBEV collected at 16 hpi, following auxin induction (1 mM) at -2 hpi, measured using RT-qPCR. TBEV RNA was normalized to actin RNA and auxin uninduced cells. Data presented as mean+SD (n=9 from 3 biological replicates). P value calculated by two-way ANOVA using Šídák's multiple comparisons test.

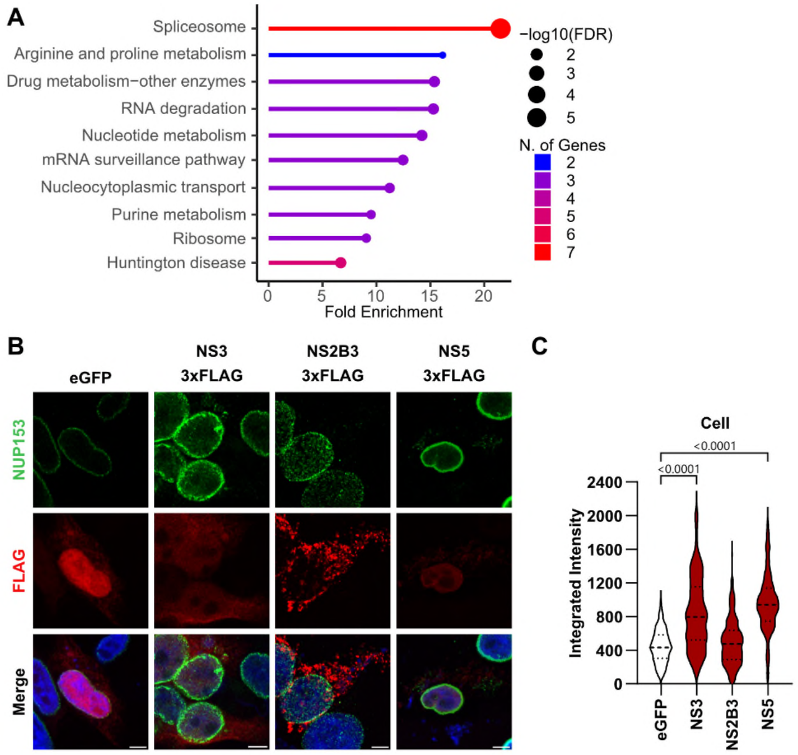

Supplementary Figure 3: NUP153 proteome is altered with infection. (A) KEGG pathway analysis of proteins found only or enriched in NUP153 mock-treated samples from NUP153 mass spectrometry pull down experiment using ShinyGO 0.80 (FDR cutoff 0.05). **(B)** Immunofluorescence analysis of HEK293T cells transfected with eGFP or viral proteins (NS3, NS2B3 and NS5) 3xFLAG tagged. DAPI (blue), anti-NUP153 (green) and eGFP or anti-FLAG (red). Images acquired with confocal microscope. Scale bars, 5 µm. **(C)** Quantification of NUP153 integrated intensity in whole cell. Data are presented as violin plot (n=269 [eGFP], n=208 [NS3], n=244 [NS2B3], and n=200 [NS5]). P value calculated with a one-way ANOVA using Šídák's multiple comparisons test.

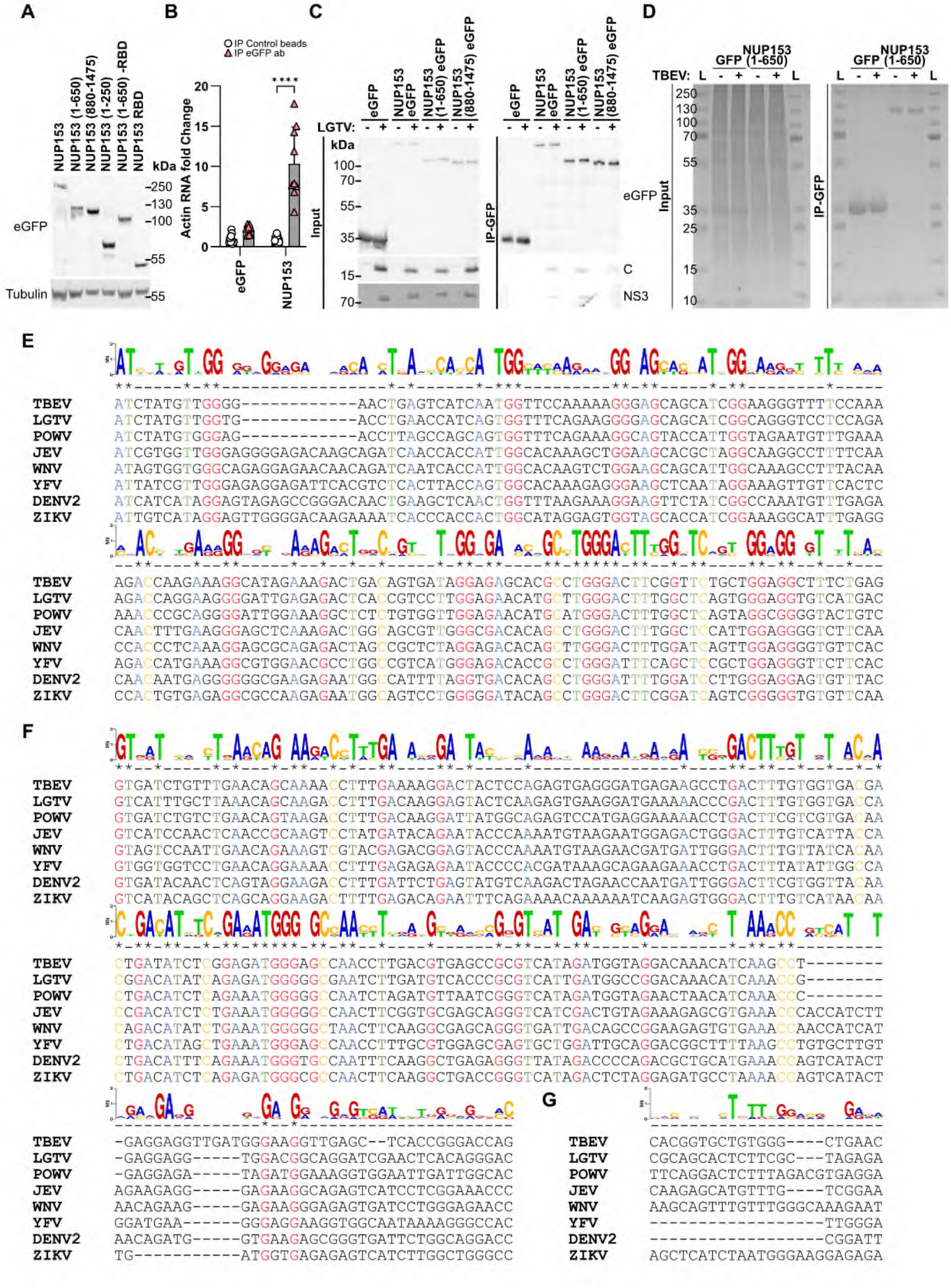

Supplementary Figure 4: N-terminal domain of NUP153 interacts with NS3 and viral RNA (A) HEK293T cells transfected with different eGFP-NUP153 truncation mutants, analyzed by immunoblotting using anti-eGFP and anti-tubulin antibodies (representative blot

of 2 independent experiments). **(B)** Crosslinking immunoprecipitation of HEK293T cells expressing eGFP-NUP153, at 48 hpi infected with LGTV. cDNA synthesis was performed using random primers. Bound actin RNA was detected using RT-qPCR. Immunoprecipitated actin RNA was normalized to input actin RNA and to samples immunoprecipitated with control beads. Bars represent means+SD (n=9 from 3 biological replicates). ****, P<0.0001; calculated by two-tailed Student's *t* test. **(C)** Co-immunoprecipitation analysis of HEK293T cells transfected with plasmids encoding eGFP-NUP153, eGFP-NUP153 truncation mutants or eGFP, and infected with LGTV for 48 hpi. Whole-cell lysates (Input) and immunoprecipitates (IP) were analyzed by immunoblotting using anti-eGFP, anti-C and anti-NS3 (Representative blot of 3 independent experiments). **(D)** CLIP-seq lysates (input) and immunoprecipitated (IP-GFP) samples PageBlue stained SDS-Page gel of HEK293T cells transfected with eGFP-$NUP153_{1\text{-}650}$ and eGFP infected with TBEV for 48 h followed by GFP-Trap pull down. L (Ladder) (PageRuler^{TM} Plus prestained protein ladder, 10 to 250 kDa – Thermo Fisher Scientific). **(E-G)** Orthoflavivirus multiple sequence alignment of peaks identified in CLIP-seq experiment. Peak 1 found in E **(E)**, peak 2 found in NS3 **(F)** and peak 3 found in NS4B **(G)** coding regions respectively**.** Orthoflavivirus sequences were aligned using AliView[83] and consensus sequence made with weblogo[92].

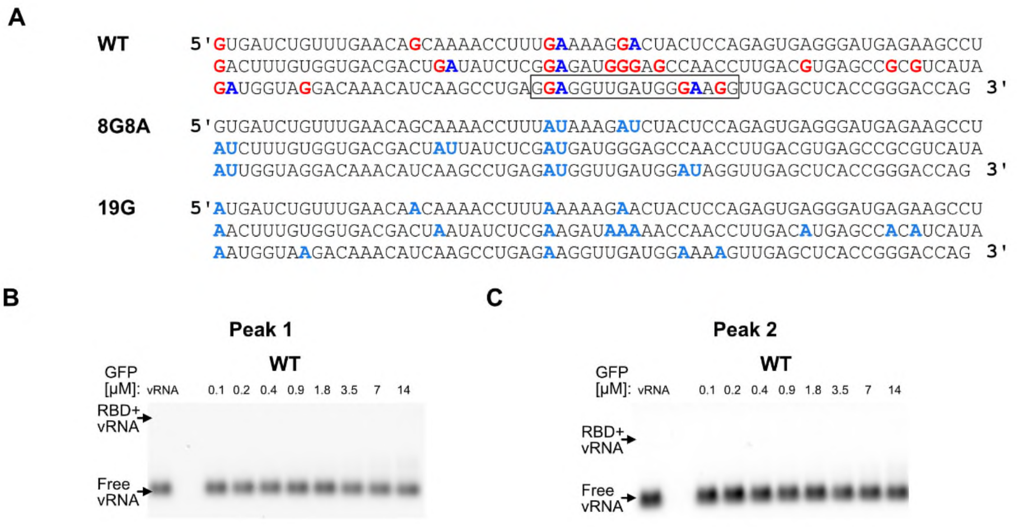

Supplementary Figure 5: GFP does not interact with NUP153-viral RNA interacting regions. (A) RNA sequence of peak 2 identified from CLIP-seq experiment (wildtype [WT]) and mutant fragments 8G8A and 19G. Orthoflavivirus-conserved guanines are red and conserved adenines in dark blue, and changes made in the mutants are light blue. Predicted G4 is enclosed in a box. **(B-C)** RNA EMSA of Cy3 tagged fragments of wild type or mutated constructs of peak 1 [2118-2264] **(B)** and peak 2 [5760-5948] **(C)** and indicated concentrations of purified His-eGFP protein.

A

Peak	Location	Position	Length	G-Quadruplex sequence	G-Score
1	E	2147	30	GGUUCCAAAAAGGGAGCAGCAUCGGAAGGG	14
1	E	2235	21	GGACUUCGGUUCUGCUGGAGG	15
2	NS3	5913	17	GGAGGUUGAUGGGAAGG	17

B

WT Long

5' CAGUCAGGGCAGUGGCACAUGGAUCUCCAGAUGUGAACGUGGCCAUGCUGAUAACGCCAAACCCAACAAUUGAA
AAUAAUGGGGGUGGCUUCAUAGAGAUGCAGCUGCCCCCAGGGGACAACAUC**AUCUAUGUUGGGGAACUGAGUCA**
UCAAUGGUUCCAAAAAGGGAGCAGCAUCGGAAGGGUUUUCCAAAAGACCAAGAAAGGUAUAGAAAGACUGACAG
UGAUAGGAGAGCACGCCUGGGACUUCGGUUCUGCUGGAGGCUUUCUGAGUUCAAUUGGGAAGGCGGUGCACACG
GUCCUUGGUGGUGCUUUCAACAGCAUCUUCGGGGGAGUGGGGUUUCUACCAAAGCUUUUAUUAGGAGUGUCAUU
GGCUUGGUUGGGCCUGAACAUGAGAAACCCUACAAUGUCCAUGAGCUUUCUCUUGGCUGGAGGCCUGGUCUUGG
CCAUGACCCUUGGAGUGGGGGCGGAUGUUGGCUGCGCUGUGGACACGGAACGAAUGGAGCUCCGCUGUGGCGAG
GGCCUGGUCGUGUGGAGAGAGGUCUCAGAAUGGUAUGACAACUAUGCCUACUACCCGGAGACACCGG 3'

11G1C Long

5' CAGUCAGGGCAGUGGCACAUGGAUCUCCAGAUGUGAACGUGGCCAUGCUGAUAACGCCAAACCCAACAAUUGAA
AAUAAUGGGGGUGGCUUCAUAGAGAUGCAGCUGCCCCCAGGGGACAACAUC**AUCUAUGUUGGGGAACUGAGUCA**
UCAAUGGUUCCAAAAAGGGAGCAGCAUCGGAAGGGUUUUCCAAAAGACCAAGAAAGGUAUAGAAAGACUGACAG
UGAUAAUACAACACAUCUACAACUUCAUUUCUGCUAUAAUCUUUCUGAGUUCAAUUGGGAAGGCGGUGCACACG
GUCCUUGGUGGUGCUUUCAACAGCAUCUUCGGGGGAGUGGGGUUUCUACCAAAGCUUUUAUUAGGAGUGUCAUU
GGCUUGGUUGGGCCUGAACAUGAGAAACCCUACAAUGUCCAUGAGCUUUCUCUUGGCUGGAGGCCUGGUCUUGG
CCAUGACCCUUGGAGUGGGGGCGGAUGUUGGCUGCGCUGUGGACACGGAACGAAUGGAGCUCCGCUGUGGCGAG
GGCCUGGUCGUGUGGAGAGAGGUCUCAGAAUGGUAUGACAACUAUGCCUACUACCCGGAGACACCGG 3'

C

	Length	Fragment	G%	Total G	Conserved G%
Peak 1	146 nt	WT	30.8	45	55.6
		11G1C	22.6	34	31.1
		28G	11.6	17	0
Peak 2	188 nt	WT	31.9	60	31.1
		8G8A	27.7	52	18.3
		19G	21.8	41	0

Supplementary Figure 6: G4 in viral RNA peaks interacting with NUP153. (A) Table listing predicted G4 in peaks 1 and 2 using QGRS mapper[43]. G4s are underlined in RNA sequence. **(B)** Sequence of RNA fragments used for G4 immunofluorescence transfection assay (length 585 nt). Peak 1 location in RNA fragment is marked in bold. Orthoflavivirus-conserved guanines are denoted in red and mutations are in blue. Predicted G4s are encased in boxes. **(C)** Table listing the length, guanine (G) content and conserved Gs in the different tested RNA fragments from peak 1 and peak 2.

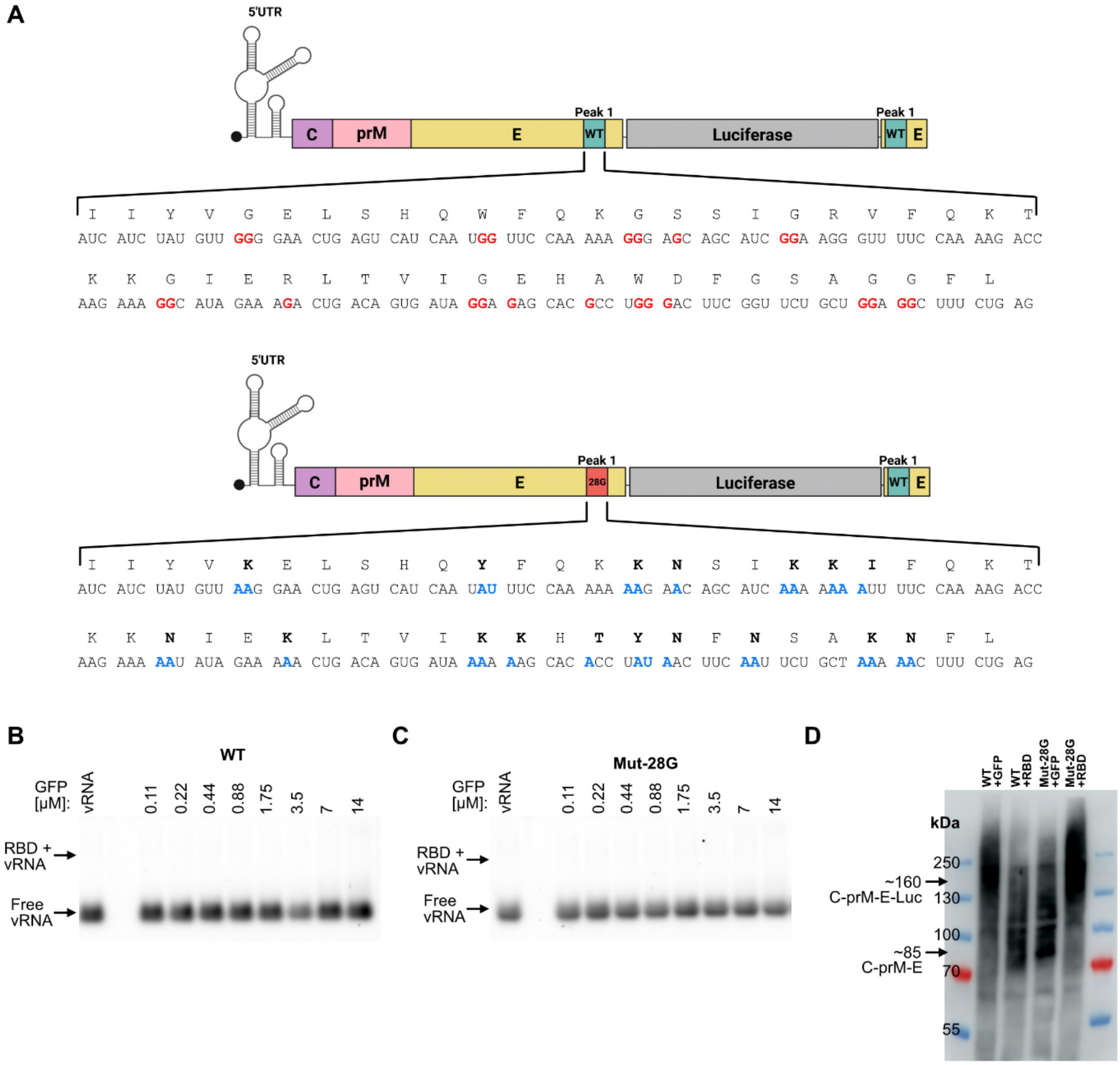

Supplementary Figure 7: In vitro translation assay of luciferase constructs in the presence or absence of NUP153 RBD. (A) Schematic representation of mRNA constructs used in in vitro translation assay. Orthoflavivirus conserved Gs are marked red and mutations are marked in blue. Amino acid changes are marked in bold. **(B-C)** RNA EMSA of Cy3 tagged fragments of wild type or mutated constructs described in **A** and indicated concentrations of purified His-eGFP protein. **(D)** Representative western blot of in vitro translation assay of WT and Mut-28G RNA treated with His-eGFP or His-eGFP-NUP153$_{RBD}$ protein detecting for anti-E antibody (Representative blot of 3 independent experiments).

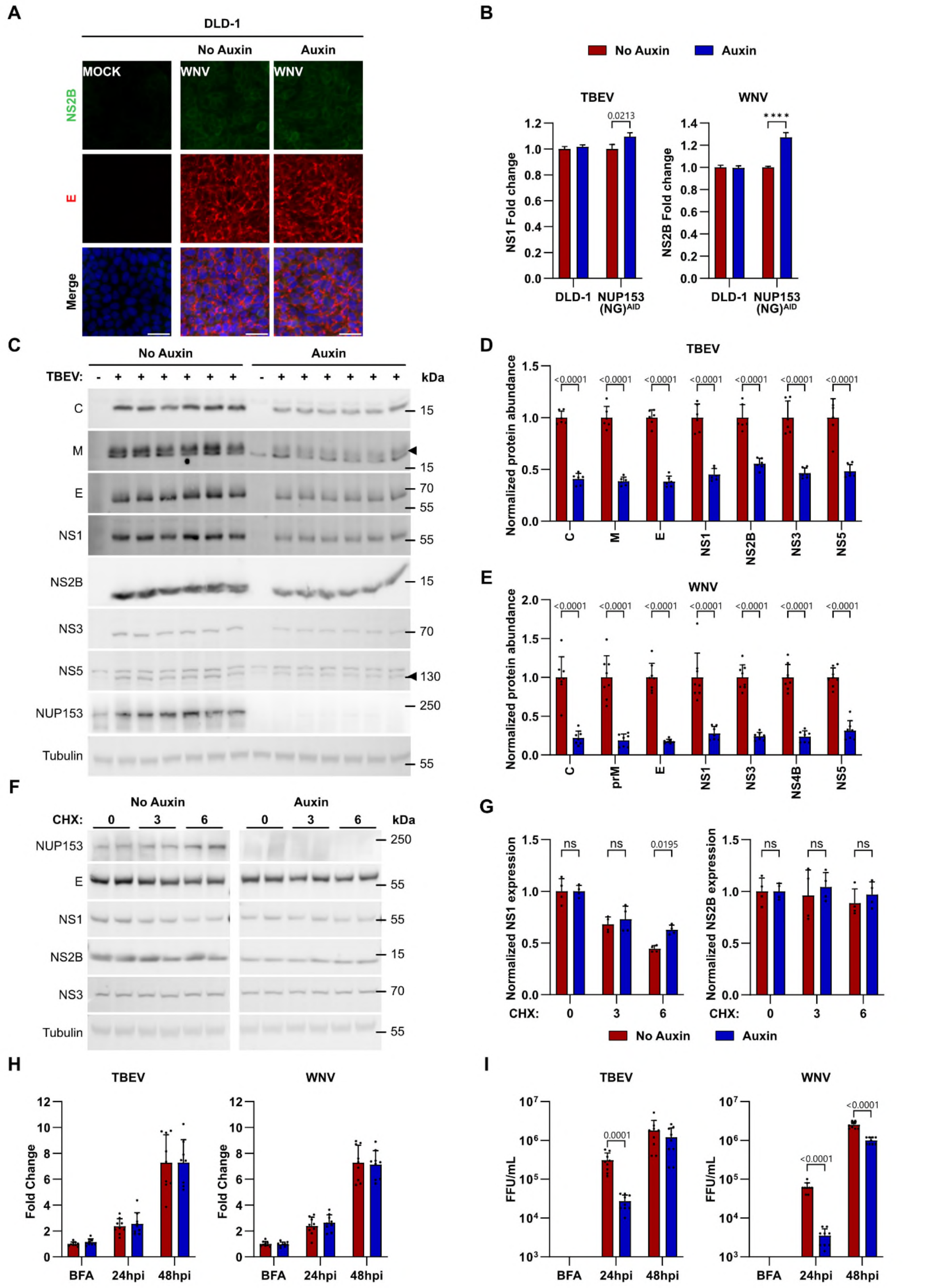

Supplementary Figure 8: NUP153 regulates viral structural to nonstructural protein ratio during orthoflavivirus infection. (A) Representative images of WNV-infected DLD-1 cells

stained with DAPI (blue), anti-WNV-NS2B antibodies (green), and anti-panFlavi-E antibodies (red), acquired using Cytation 5 system. Scale bars, 30 µm. **(B)** Analysis of viral protein NS1 (TBEV) or NS2B (WNV) levels in DLD-1 and NUP153(NG)^{AID} infected cells, ±auxin 2 h prior to infection with TBEV (analysis at 16 hpi) or WNV (analysis at 48 hpi). **(C)** Western blots using indicated antibodies used for quantification in Fig 8H and Supp Fig 8D. Arrows indicate correct bands for antibodies where more than one band is present. Each blot shows all the biological replicas n=6. **(D-E)** Total amount of detected TBEV (quantified by western blot) **(D)** or WNV (quantified by mass spectrometry) **(E)** proteins in ±auxin samples normalized to tubulin (TBEV) or total protein abundance (WNV) and auxin uninduced samples. Data are presented as the mean+SD (TBEV n=6; WNV n=8). ****, P<0.0001; calculated by two-way ANOVA using Šídák's multiple comparisons test. **(F-G)** Viral protein stability in ±auxin NUP153(NG)^{AID} TBEV infected cells treated 24 hpi with CHX for 0, 3 or 6 hours. Representative western blots using indicated antibodies used for quantification in Fig 8J and Supp Fig 8G **(F)**. NS1 (left) and NS2B (right) protein expressions were normalized to tubulin expression in each sample and to the CHX 0 timepoint of either ±auxin cells. ns, not significant; P values calculated by two-way ANOVA using Šídák's multiple comparisons test. Data presented as mean+SD (n=4 biological replicates) **(G)**. **(H)** BFA assay on DLD-1 ±auxin 2 h prior to infection with TBEV or WNV. Cells were treated with BFA 16 hpi. E positive cells were quantified using a plate reader. Samples were normalized to BFA treated auxin uninduced cells. Error bars represent mean+SD (n=9 from 3 biological replicates). **(I)** Viral titers of BFA assay of NUP153(NG)^{AID} cells ±auxin 2 h prior to infection with TBEV or WNV. Cells were treated with BFA at 16 hpi. Data are presented as the mean+SD (n=9 from 3 biological replicates). P values calculated by two-tailed Student's *t* test.

Supplemental Tables

Supp Table 1: Antibodies used in this study.

Antibodies	Origin	Clonality	Working dilution	Company (Cat#)
Anti-mouse HRP	Goat	Polyclonal	1:1000 (FFA)	Thermo Fisher Scientific (31430)
Anti-rabbit HRP	Goat	Polyclonal	1:5000 (WB)	Thermo Fisher Scientific (31460)
Anti-rabbit Alexa Fluor 488	Donkey	Polyclonal	1:500 (IF), 1:2500 (WB)	Thermo Fisher Scientific (A21206)
Anti-rabbit Alexa Fluor 555	Donkey	Polyclonal	1:500 (IF), 1:2500 (WB)	Thermo Fisher Scientific (A31572)
Anti-rabbit Alexa Fluor 647	Donkey	Polyclonal	1:500 (IF), 1:2500 (WB)	Thermo Fisher Scientific (A31573)
Anti-mouse Alexa Fluor 488	Donkey	Polyclonal	1:500 (IF), 1:2500 (WB)	Thermo Fisher Scientific (A21202)
Anti-mouse Alexa Fluor 555	Donkey	Polyclonal	1:500 (IF), 1:2500 (WB)	Thermo Fisher Scientific (A31570)
Anti-mouse Alexa Fluor 647	Donkey	Polyclonal	1:500 (IF), 1:2500 (WB)	Thermo Fisher Scientific (A31571)
Anti-chicken Alexa Fluor 555	Goat	Polyclonal	1:500 (IF), 1:2500 (WB)	Thermo Fisher Scientific (A21437)
Anti-chicken Alexa Fluor 647	Goat	Polyclonal	1:500 (IF), 1:2500 (WB)	Thermo Fisher Scientific (A21449)
Anti-FLAG M2	Mouse	Monoclonal	1:500 (IF)	Stratagene (200472)
Anti-GAPDH	Rabbit	Polyclonal	1:500 (IF), 1:2000(WB)	Sigma (G9545)
Anti-eGFP	Rabbit	Polyclonal	1:2000 (WB)	Thermo Fisher Scientific (A6455)
Anti-Tubulin	Rabbit	Polyclonal	1:4000 (WB)	Abcam (ab6046-100)
Anti-Tubulin hFAB Rhodamine			1:4000 (WB)	BioRad (12004166)
Anti-mab414	Mouse	Monoclonal	1:500 (IF)	Abcam (ab24609)
Anti-NUP153	Rabbit	Polyclonal	1:500 (IF), 1:1000(WB)	Abcam (ab84872)
Anti-Calnexin	Rabbit	Polyclonal	1:500 (IF)	Abcam (ab75801)
Anti-G4	Mouse	Monoclonal	1:300 (IF)	Sigma (ZMS1070)
Anti-dsRNA(J2)	Mouse	Monoclonal	1:1000 (IF)	Scicons (10010500)
Anti-panFlaviE	Mouse	Monoclonal	1:1000 (IF)	NovusBio (NBP2-52709)
Anti-WNV_NS2B	Rabbit	Polyclonal	1:500 (IF)	GeneTex (GTX132060)
Anti-TBEV_C	Rabbit	Polyclonal	1:1000 (WB)	[84]
Anti-TBEV_M	Rabbit	Polyclonal	1:1000 (WB)	[85]
Anti-TBEV_E_1783.3	Mouse	Monoclonal	1:1000 (IF)	Gift from Matthias Niedrig[86]
Anti-TBEV_E	Rabbit	Monoclonal	1:1000 (WB)	GeneTex (GTX642362)
Anti-TBEV_E	Rabbit	Monoclonal	1:1000 (WB)	GeneTex (GTX642364)
Anti-TBEV_NS1	Chicken	Polyclonal	1:500 (IF)	[87]
Anti-TBEV_NS1	Rabbit	Monoclonal	1:1000 (WB)	GeneTex (GTX642144)
Anti-TBEV_NS2B	Rabbit	Monoclonal	1:1000 (WB)	GeneTex (GTX642952)
Anti-TBEV_NS3	Chicken	Polyclonal	1:500 (IF), 1:1500 (WB)	[2]
Anti-TBEV_NS5	Chicken	Polyclonal	1:500 (IF), 1:1000 (WB)	[88]
Anti-TBEV_NS5	Rabbit	Monoclonal	1:1000 (WB)	GeneTex (GTX642476)
APC Conjugation Kit Lightning-Link®				Abcam (ab201807)

Supp Table 2: Primers used for RT-qPCR

Primer	Sequence (5´-3´)	5´ dye	3’
LGTV NS3 Forward [76]	AACGGAGCCATAGCCAGTGA		
LGTV NS3 Reverse [76]	AACCCGTCCCGCCACTC		
LGTV NS3 Probe [76]	AGAGACAGATCCCTGATGG	FAM6	BHQ
TBEV Forward [89]	GGGCGGTTCTTGTTCTCC		
TBEV Reverse [89]	ACACATCACCTCCTTGTCAGACT		
TBEV Probe [89]	TGAGCCACCATCACCCAGACACA	FAM6	BHQ
NUP153 Forward [14]	AGCCTGTGAAACACCGAAAC		
NUP153 Reverse [14]	AGCTGGAAGATGAAGCAGTCA		
