# Supplementary material for: Proviral NUP153 binding to viral proteins and RNA regulates structural–nonstructural protein ratios in orthoflavivirus infection
Source: Nat Commun. 2026 Apr 8;17:3402. doi: 10.1038/s41467-026-71449-1 (PMC13068965; doi:10.1038/s41467-026-71449-1)
Supplement: Supplementary file 2 — Description of Additional Supplementary Files [file 41467_2026_71449_MOESM2_ESM.pdf]

<u>Description of Additional Supplementary Files</u>

Supplemental Data 1:

Proteins identified in the mass spectrometry analysis.
