# Supplementary material for: Proviral NUP153 binding to viral proteins and RNA regulates structural–nonstructural protein ratios in orthoflavivirus infection
Source: Nat Commun. 2026 Apr 8;17:3402. doi: 10.1038/s41467-026-71449-1 (PMC13068965; doi:10.1038/s41467-026-71449-1)
Supplement: Supplementary file 4 — Reporting Summary [file 41467_2026_71449_MOESM4_ESM.pdf]

nature portfolio

Corresponding author(s): Anna Överby

Last updated by author(s): Mar 3, 2026

Reporting Summary

Nature Portfolio wishes to improve the reproducibility of the work that we publish. This form provides structure for consistency and transparency in reporting. For further information on Nature Portfolio policies, see our Editorial Policies and the Editorial Policy Checklist.

Statistics

For all statistical analyses, confirm that the following items are present in the figure legend, table legend, main text, or Methods section.

n/a	Confirmed	
☐	☒	The exact sample size (n) for each experimental group/condition, given as a discrete number and unit of measurement
☐	☒	A statement on whether measurements were taken from distinct samples or whether the same sample was measured repeatedly
☐	☒	The statistical test(s) used AND whether they are one- or two-sided<br>*Only common tests should be described solely by name; describe more complex techniques in the Methods section.*
☒	☐	A description of all covariates tested
☒	☐	A description of any assumptions or corrections, such as tests of normality and adjustment for multiple comparisons
☐	☒	A full description of the statistical parameters including central tendency (e.g. means) or other basic estimates (e.g. regression coefficient) AND variation (e.g. standard deviation) or associated estimates of uncertainty (e.g. confidence intervals)
☐	☒	For null hypothesis testing, the test statistic (e.g. F, t, r) with confidence intervals, effect sizes, degrees of freedom and P value noted<br>*Give P values as exact values whenever suitable.*
☒	☐	For Bayesian analysis, information on the choice of priors and Markov chain Monte Carlo settings
☒	☐	For hierarchical and complex designs, identification of the appropriate level for tests and full reporting of outcomes
☒	☐	Estimates of effect sizes (e.g. Cohen's d, Pearson's r), indicating how they were calculated

Our web collection on statistics for biologists contains articles on many of the points above.

Software and code

Policy information about availability of computer code

Data collection	Amersham Imager 680 (System has an integrated analysis software)<br>BioTek Gen5 software (v3.16.10)<br>Leica Application Suit X software (LAS X, v.3.5.5)<br>StepOnePlus real-time PCR system (v2.3)
Data analysis	AliView (v1.28)<br>Bedtools (v2.30.0)<br>BioTek Gen5 software (v3.16.10)<br>Bowtie (v1.3.1)<br>Bowtie2 (v.2.4.4)<br>CellProfiler (v4.2.5)<br>Cutadapt (v3.5)<br>FastQC (v0.11.9)<br>GraphPad Prism (v10.2.3)<br>ImageJ (v1.54)<br>Imaris (10.2)<br>Leica Application Suit X software (LAS X, v.3.5.5)<br>MaxQuant (v2.0.1.0)<br>PEAKachu (Galaxy Version 0.2.0)<br>Perseus (v2.03.0)<br>RStudio Desktop (v2023.09.1)

Samtools (v1.13)
ShinyGO (v0.80)

For manuscripts utilizing custom algorithms or software that are central to the research but not yet described in published literature, software must be made available to editors and reviewers. We strongly encourage code deposition in a community repository (e.g. GitHub). See the Nature Portfolio guidelines for submitting code & software for further information.

Data

Policy information about availability of data

All manuscripts must include a data availability statement. This statement should provide the following information, where applicable:

- Accession codes, unique identifiers, or web links for publicly available datasets
- A description of any restrictions on data availability
- For clinical datasets or third party data, please ensure that the statement adheres to our policy

All data supporting the findings of this study is provided within the article. Raw data from AP-MS and MS has been deposited in the PRIDE repository with identifier PXD052523 and PXD067546 respectively. Raw data from CLIP-seq experiment generated in this study has been deposited Sequence Read Archive database with accession code PRJNA1137903. Viral genome sequences are publicly accessible in GeneBank: TBEV (DQ401140.3), LGTV (EU790644.1), POWV (NC_003687.1), JEV (NC_001437.1), WNV (NC_009942.1), YFV (NC_002031.1), DENV2 (NC_001474.2:97-10272) and ZIKV (NC012532.1).

Research involving human participants, their data, or biological material

Policy information about studies with human participants or human data. See also policy information about sex, gender (identity/presentation), and sexual orientation and race, ethnicity and racism.

Reporting on sex and gender	Not relevant
Reporting on race, ethnicity, or other socially relevant groupings	Not relevant
Population characteristics	Not relevant
Recruitment	Not relevant
Ethics oversight	Not relevan

Note that full information on the approval of the study protocol must also be provided in the manuscript.

Field-specific reporting

Please select the one below that is the best fit for your research. If you are not sure, read the appropriate sections before making your selection.

☒ Life sciences ☐ Behavioural & social sciences ☐ Ecological, evolutionary & environmental sciences

For a reference copy of the document with all sections, see nature.com/documents/nr-reporting-summary-flat.pdf

Life sciences study design

All studies must disclose on these points even when the disclosure is negative.

Sample size	No sample size calculation was performed using statistical methods. Sample sizes were chosen based on similar studies performed previously. Sample size used in each experiment was included in the figure legends. Immunofluorescence experiments were replicated three times acquiring 9 images (Stiched) each time per replicate for each condition. Data was analyzed on a per-cell basis. The number of cells was not predetermined and varies depending on the number of cells in frame of the microscopy picture.
Data exclusions	No data points were excluded from the datasets in this study.
Replication	The experimental results found in this study were reliably and consistently reproducible. The number of replicates and data points can be found in the figure legends. Minimum three biological replicates were performed for CoIP, CLIP, Immunofluorecense and plate reader immunofluorecense experiments. A minimum of two biological replicates were performed for the in-vitro binding assay. Biological replicates were performed on different days. For all experiments excluding western blots, technical replicates were included for each biological replicate. AP-MS and CLIP-seq both contain three independent replicates. MS on NUP153-(NG)AID cells contain eight independent replicates.
Randomization	Randomization was not necessary for the experiments performed in this work.
Blinding	Blinding was not necessary for the experiments included in this work

Reporting for specific materials, systems and methods

We require information from authors about some types of materials, experimental systems and methods used in many studies. Here, indicate whether each material, system or method listed is relevant to your study. If you are not sure if a list item applies to your research, read the appropriate section before selecting a response.

Materials & experimental systems

n/a	Involved in the study
☐	☒ Antibodies
☐	☒ Eukaryotic cell lines
☒	☐ Palaeontology and archaeology
☒	☐ Animals and other organisms
☒	☐ Clinical data
☒	☐ Dual use research of concern
☒	☐ Plants

Methods

n/a	Involved in the study
☒	☐ ChIP-seq
☒	☐ Flow cytometry
☒	☐ MRI-based neuroimaging

Antibodies

Antibodies used

All antibodies used including species, dilutions and cat numbers were provided in the Supplementary table 2.

Validation

All antibodies have been validated by the manufacturers or in other publications.

https://www.thermofisher.com/antibody/product/Goat-anti-Mouse-IgG-H-L-Secondary-Antibody-Polyclonal/31430
https://www.thermofisher.com/antibody/product/Donkey-anti-Rabbit-IgG-H-L-Highly-Cross-Adsorbed-Secondary-Antibody-Polyclonal/A-21206
https://www.thermofisher.com/antibody/product/Donkey-anti-Rabbit-IgG-H-L-Highly-Cross-Adsorbed-Secondary-Antibody-Polyclonal/A-31572
https://www.thermofisher.com/antibody/product/Donkey-anti-Rabbit-IgG-H-L-Highly-Cross-Adsorbed-Secondary-Antibody-Polyclonal/A-31573
https://www.thermofisher.com/antibody/product/Donkey-anti-Mouse-IgG-H-L-Highly-Cross-Adsorbed-Secondary-Antibody-Polyclonal/A-21202
https://www.thermofisher.com/antibody/product/Donkey-anti-Mouse-IgG-H-L-Highly-Cross-Adsorbed-Secondary-Antibody-Polyclonal/A-31570
https://www.thermofisher.com/antibody/product/Donkey-anti-Mouse-IgG-H-L-Highly-Cross-Adsorbed-Secondary-Antibody-Polyclonal/A-31571
https://www.thermofisher.com/antibody/product/Goat-anti-Chicken-IgY-H-L-Secondary-Antibody-Polyclonal/A-21437
https://www.thermofisher.com/antibody/product/Goat-anti-Chicken-IgY-H-L-Secondary-Antibody-Polyclonal/A-21449
https://www.sigmaaldrich.com/SE/en/product/sigma/g9545
https://www.thermofisher.com/antibody/product/GFP-Antibody-Polyclonal/A-6455
https://www.abcam.com/en-se/products/primary-antibodies/beta-tubulin-antibody-loading-control-ab6046#
https://www.abcam.com/en-se/products/primary-antibodies/nuclear-pore-complex-proteins-antibody-mab414-ab24609#
https://www.abcam.com/en-se/products/primary-antibodies/nup153-antibody-ab84872
https://www.abcam.com/en-se/products/primary-antibodies/calnexin-antibody-er-membrane-marker-ab75801
https://www.sigmaaldrich.com/SE/en/product/sigma/zms1070
https://www.exalpha.com/products/scicons-anti-dsrna-antibody-j2/10010500
https://www.novusbio.com/products/flavivirus-group-antigen-antibody-d1-4g2-4-15-4g2-_nbp2-52709
https://www.genetex.com/Product/Detail/West-Nile-virus-NS2B-protein-antibody/GTX132060
https://www.genetex.com/Product/Detail/Tick-borne-encephalitis-virus-TBEV-Envelope-antibody-HL4031/GTX642362
https://www.genetex.com/Product/Detail/Tick-borne-encephalitis-virus-NS2B-antibody-HL4147/GTX642952
https://www.genetex.com/Product/Detail/Tick-borne-encephalitis-virus-NS5-antibody-HL4061/GTX642476

In house produced antibodies have previously been validated in other publications. Furthermore all viral antibodies were validated with staining of mock treated samples as shown at different points in this study.

Anti-TBEV_C: Vonderstein, K. et al. Viperin Targets Flavivirus Virulence by Inducing Assembly of Noninfectious Capsid Particles. J Virol 92 (2018). https://doi.org:10.1128/JVI.01751-17
Anti-TBEV_E_1783.3: Niedrig, M. et al. Monoclonal antibodies directed against tick-borne encephalitis virus with neutralizing activity in vivo. Acta virologica 38, 141-149 (1994)
Anti-TBEV_NS1: Yau, W. L. et al. Model System for the Formation of Tick-Borne Encephalitis Virus Replication Compartments without Viral RNA Replication. Journal of virology 93 (2019). https://doi.org:10.1128/JVI.00292-19
Anti-TBEV_NS3: Panayiotou, C. et al. Viperin Restricts Zika Virus and Tick-Borne Encephalitis Virus Replication by Targeting NS3 for Proteasomal Degradation. J Virol 92 (2018). https://doi.org:10.1128/JVI.02054-17
Anti-TBEV_NS5: Chotiwan, N. et al. Type I interferon shapes brain distribution and tropism of tick-borne flavivirus. Nat Commun 14, 2007 (2023). https://doi.org:10.1038/s41467-023-37698-0

Eukaryotic cell lines

Policy information about cell lines and Sex and Gender in Research

Cell line source(s)	A549: Kind Gift of Firedemann Weber, Department of Virology, University of Freiburg, Freiburg, Germany<br>HEK293T: Kind Gift of Firedemann Weber, Department of Virology, University of Freiburg, Freiburg, Germany<br>VeroB4: Kind gift of Gerhard Dobler Bundeswehr Institute of Microbiology, Munich, Germany.<br>DLD-1: Kind gift of Mary Dasso Division of Molecular and Cellular Biology, National Institute of Child Health and Human Development, National Institutes of Health, Bethesda, MD, 20892, USA.<br>DLD-1 NUP153(NG)AID: Kind gift of Mary Dasso Division of Molecular and Cellular Biology, National Institute of Child Health and Human Development, National Institutes of Health, Bethesda, MD, 20892, USA.
Authentication	None of the cell lines were in-house authenticated.
Mycoplasma contamination	The cells were not contaminated with mycoplasm tested by MycoAlert (Lonza, Cat # LT07-318)
Commonly misidentified lines<br>(See ICLAC register)	No commonly misidentified lines according to the ICLAC register were used

Plants

Seed stocks	*Report on the source of all seed stocks or other plant material used. If applicable, state the seed stock centre and catalogue number. If plant specimens were collected from the field, describe the collection location, date and sampling procedures.*
Novel plant genotypes	*Describe the methods by which all novel plant genotypes were produced. This includes those generated by transgenic approaches, gene editing, chemical/radiation-based mutagenesis and hybridization. For transgenic lines, describe the transformation method, the number of independent lines analyzed and the generation upon which experiments were performed. For gene-edited lines, describe the editor used, the endogenous sequence targeted for editing, the targeting guide RNA sequence (if applicable) and how the editor was applied.*
Authentication	*Describe any authentication procedures for each seed stock used or novel genotype generated. Describe any experiments used to assess the effect of a mutation and, where applicable, how potential secondary effects (e.g. second site T-DNA insertions, mosiacism, off-target gene editing) were examined.*
